# Dementia-related disability in the population aged 90 years and over: differences over time and the role of comorbidity in the vitality 90 + study

**DOI:** 10.1186/s12877-023-03980-5

**Published:** 2023-05-06

**Authors:** Saritha Susan Vargese, Marja Jylhä, Jani Raitanen, Linda Enroth, Pauliina Halonen, Mari Aaltonen

**Affiliations:** 1 Faculty of Social Sciences (Health Sciences) and Gerontology Research Center (GEREC), Tampere University Hospital, Tampere, Finland; 2 Believers Church Medical College Hospital, Thiruvalla, India; 3 The UKK Institute for Health Promotion Research, Tampere, Finland; 4 Finnish Institute for Health and Welfare, Helsinki, Finland

**Keywords:** Physical functioning, Oldest old, Comorbidity, Memory disorders

## Abstract

**Background:**

The burden of dementia, multimorbidity, and disability is high in the oldest old. However, the contribution of dementia and comorbidities to functional ability in this age group remains unclear. We examined the combined effects of dementia and comorbidities on ADL and mobility disability and differences between dementia-related disability between 2001, 2010, and 2018.

**Methods:**

Our data came from three repeated cross-sectional surveys in the population aged 90 + in the Finnish Vitality 90 + Study. The associations of dementia with disability and the combined effects of dementia and comorbidity on disability adjusted for age, gender, occupational class, number of chronic conditions, and study year were determined by generalized estimating equations. An interaction term was calculated to assess differences in the effects of dementia on disability over time.

**Results:**

In people with dementia, the odds of ADL disability were almost five-fold compared to people with three other diseases but no dementia. Among those with dementia, comorbidities did not increase ADL disability but did increase mobility disability. Differences in disability between people with and without dementia were greater in 2010 and 2018 than in 2001.

**Conclusion:**

We found a widening gap in disability between people with and without dementia over time as functional ability improved mainly in people without dementia. Dementia was the main driver of disability and among those with dementia, comorbidities were associated with mobility disability but not with ADL disability. These results imply the need for strategies to maintain functioning and for clinical updates, rehabilitative services, care planning, and capacity building among care providers.

**Supplementary Information:**

The online version contains supplementary material available at 10.1186/s12877-023-03980-5.

## Introduction

The risks of dementia and disability are higher in the oldest old than in younger old people and are known to increase even in very old age [1, 2]. The concept of the oldest old varies between studies, but is usually defined as persons aged 80, 85, or 90 years and older [3]. Nearly 40% of people over 90 suffer from dementia, and studies have found an incidence rate of 14 per 100 person years [4]. In all, dementia is common condition among the older adults [1]. People with dementia are more likely to perform poorly in activities of daily living (ADL) [5] and mobility [6, 7] than those without dementia, and the level of disability increases as the disease progresses [7]. Recent studies report improving trends in functioning among the oldest old, likely due to better living conditions, improved medical care and physical and technological support [8–10], but the differences in disability in people with and without dementia over time are not well-established.

Multimorbidity may play a role in the association between dementia and functional disability. Multimorbidity refers to a situation where at least two conditions coexist without any one predominant condition, while comorbidity refers to extra coexisting conditions with an index disease [11]. In persons aged 70–80 years, disability was found to increase with the number of chronic conditions co-existing with dementia [12, 13]. In people over 90 years, both dementia and multimorbidity increased the risk for care home admission and mortality [14]. Functional disability in older adults, with considerable individual differences [15], is associated with higher health care utilization and costs, institutionalization [16], and mortality [17].

The contribution of dementia to functional disability and need for care in older people is bound to increase in the future. Although it has been suggested that the incidence [18] and prevalence [19] of dementia are on the decline in Europe, the proportion of people living with dementia is projected to double from 1.6% in 2018 to 3% in 2050 due to its strong association with age and the growth of the older population, especially those over 85 years, in the decades ahead [19]. In Finland, the number of people aged 90 or over has doubled from 2000 to 2015 and is projected to double yet again by 2035 [20]. In the United States, this group is anticipated to quadruple in size from 2000 to 2040, and at the same time its proportion of the total population will rise from 0.5% to 1.6% [21]. The increasing impact of dementia on care needs, especially on the need for long-term care, is well known [14, 22]. However, research on physical disability and dementia in the very old population is still scarce. As people with dementia also are living longer than before, and the comorbidity of dementia is changing [23, 24], this study set out to explore whether the effect of dementia and comorbidities on disability has changed over time.

The study focuses on the associations of dementia and comorbidity with disability in the oldest old population using data from three repeated surveys conducted with the exact same methods. We have three research questions: [1] to what extent is dementia associated with ADL and mobility disability in the oldest old, [2] what is the combined association of dementia and comorbidities with ADL and mobility disability, and [3] to what extent does the association of dementia and comorbidities with ADL and mobility disability differ between 2001, 2010, and 2018?

## Methods

This study is part of the population-based Vitality 90 + Study, a multidisciplinary research project with nonagenarians conducted in Tampere, Finland [10, 25], where in 2019, the people aged 90 or over accounted for 0.9% of the whole population of 238,140. All individuals aged 90 or over in the area, both community-dwelling and institutionalized, were invited to participate in mailed surveys in 2001 (*N* = 1063), 2010 (*N* = 1606), and 2018 (*N* = 2449). We received 4047 responses from 3907 participants; 892 in 2001, 1277 in 2010, and 1878 in 2018; the increasing number reflects the growth of the oldest-old population. Among the respondents, 35 (0.9%) participated in 2001 and 2010, 103 (2.7%) in 2010 and 2018, and 1 (0.03%) participated in all three study years. All other participants responded only once, mainly due to high mortality. The response rates were 83.9%, 79.5%, and 76.7% in 2001, 2010, and 2018, respectively. The proportion of persons with dementia who lived in long-term care was 59.5%, 62.2%, and 54.7%, respectively. Participants were considered self-respondents if they answered the questions themselves or with help in writing. Responses were ‘proxy reported’ when someone else, most commonly a caregiver, a family member, or a friend gave the answers. The proportion of proxy respondents ranged from 15 to 23%. As the prevalence of dementia, disability, and sensory problems are high in this age group, the use of proxy respondents improved the representativeness of the study.

### Outcome variables

Two domains were considered as indicators of disability: 1) ADL disability and 2) mobility disability. In each round of the mailed survey, the questions related to ADL were [1] “Are you able to get in and out of bed?” and [2] “Are you able to dress and undress?” The questions related to mobility were [1] “Are you able to move about indoors?” [2], “Are you able to walk 400 m?”, and [3] “Are you able to use stairs?” The response options for each question were (1) yes, without difficulty, (2) yes, with difficulty, (3) only with help, and (4) not at all. Participants able to perform an activity without help (with or without difficulty) were classified as independent, while participants able to perform an activity only with help or unable to perform an activity, were classified as dependent in the respective activity. Participants were classified as having ADL disability if they were dependent in at least one of the two ADL activities. Similarly, participants were classified as having mobility disability if they were dependent in at least one of the three mobility activities [15].

### Explanatory variables

Self-reported information on chronic conditions was collected in each survey year. The questionnaire item for dementia was worded as follows: “Has a doctor told you that you have dementia, Alzheimer’s disease, or worsening of memory?” (yes/no). In addition, participants were asked about hypertension, heart disease (coronary artery disease, arrhythmia, or myocardial infarction), stroke, diabetes, osteoarthritis, hip fracture, and depression. To examine the combined effects of dementia and comorbidities on disability, participants were categorized into six groups: 1) no dementia & no other morbidities, 2) no dementia & 1–2 morbidities, 3) no dementia & at least 3 morbidities, 4) dementia & no other morbidities, 5) dementia & 1–2 morbidities, and 6) dementia & at least 3 morbidities. Occupational class was considered as a covariate because it has been shown that the risk for cognitive and functional disability varies according to social class [22]. Occupational class, based on the longest held occupation, was categorized as non-manual, manual, housewife, and unknown [26]. Age, gender, and study year were considered as covariates and were controlled for in the analysis.

The Vitality 90 + study protocol was approved by The Ethics Committee of the City of Tampere or the Regional Ethics Committee of Tampere University Hospital, depending on the study year, and written informed consent was obtained from participants or their legal representatives.

### Data analysis

Among 4047 responses obtained from 3907 participants, information on ADL was missing for 32 persons and mobility for 82 persons, and 54 did not answer the question on dementia. Hence, the analysis was done on 3961 and 3911 observations for ADL and mobility disability, respectively. Frequencies and percentages were calculated for all independent variables, and ADL and mobility disability were analysed separately for people with and without dementia. Pearson’s chi-squared test was used to examine the association of dementia status with gender, occupational class, chronic conditions, and level of disability. Mann–Whitney U test was used to analyze the difference in age between those with and without dementia in each study year.

The association between dementia and disability was determined using a generalized estimating equation (GEE) approach with a logit link and an independent ‘working’ correlation structure. This method takes into account the dependency between observations for individuals who participated more than once and will produce valid standard errors when using the robust standard error estimator [27]. The analysis was done for both ADL and mobility disability, and age, gender, occupational class, number of chronic conditions, and study year were included into the models. Odds ratios (OR) and their 95% confidence intervals (CI) were obtained from adjusted models. In model 1, we examined the association between dementia and disability, adjusted for age, gender, and study year. Occupational class was added as a covariate in model 2 and multimorbidity in model 3. Furthermore, in model 4, we fitted the interaction term to assess whether the effect of dementia on disability was different between study years. The multivariate analyses were conducted separately for ADL and mobility disability.

To examine the combined effect of dementia and comorbidity on disability, we used a GEE approach controlling for age, gender, occupational class, and study year. The analysis was done separately for the two outcome measures. The association between dementia and disability were separately analysed for each study year using binary logistic regression with ORs and 95% CIs, and the results are presented as supplementary tables. To estimate the proportion of ADL and mobility disability that could be attributed to dementia, population attributable fraction (PAF) was computed based on the GEE model using punaf module in Stata [28, 29]. Statistical analyses were done using Stata 16.1 (College Station, TX, USA) and SPSS 25 (Armonk, NY, USA), and statistical significance was set at *p* < 0.05.

## Results

### Characteristics of the study population

In all study years the majority of the participants were women. The prevalence of dementia was 42.9% in 2001, 39.7% in 2010, and 40.0% in 2018. Participants with dementia were slightly older and had at least four morbidities and depression more often than those without dementia in all study years. ADL and mobility disability were significantly higher in people with dementia compared to those without dementia (*p* < 0.001 in all three study years). The prevalence of ADL disability was 71.2%, 77.4%, 70.9% vs. 48.2%, 48.7%, 40.7% and the prevalence of mobility disability 47.0%, 46.8%, 42.2% vs. 15.2%, 9.6%, 7.9% in people with and without dementia in the three study years (Table 1).

**Table 1:** Characteristics of study participants according to presence or absence of dementia

**Characteristics**	2001 (*n* = 874)				2010 (*n* = 1263)					2018 (*n* = 1856)			
Dementia status (%)	**D + (42.9)**	**D- (57.1)**	**Total**	***p***		**D + (39.7)**	**D- (60.3)**	**Total**	***p***	**D + (40.0)**	**D- (60.0)**	**Total**	***p***
Gender^a^, n (%)													
Males	66 (17.6)	104 (20.8)	170 (19.5)	0.231		81 (16.1)	156 (20.5)	237 (18.8)	0.052	184 (24.8)	304 (27.3)	488 (26.3)	0.222
Females	309 (82.4)	395 (79.2)	704 (80.5)			421 (83.9)	605 (79.5)	1026 (81.2)		559 (75.2)	809 (72.7)	1368 (73.7)	
Age, mean ± SD^b^	92.54 ± 2.6	92.18 ± 2.5	92.34 ± 2.5	0.042		92.86 ± 2.8	92.41 ± 2.6	92.58 ± 2.7	0.003	92.82 ± 2.7	92.56 ± 2.7	92.65 ± 2.7	0.041
Occupational class^a^, n (%)													
Non-manual	94 (25.1)	155 (31.1)	249 (28.5)	0.001		184 (36.7)	336 (44.2)	520 (41.2)	0.013	321 (43.2)	569 (51.1)	890 (48.0)	0.002
Manual	160 (42.7)	234 (46.9)	394 (45.1)			235 (46.8)	337 (44.3)	572 (45.3)		364 (49.0)	452 (40.6)	816 (44.0)	
Housewives	37 (9.9)	52 (10.4)	89 (10.2)			47 (9.4)	55 (7.2)	102 (8.1)		33 (4.4)	61 (5.5)	94 (5.1)	
Unknown	84 (22.4)	58 (11.6)	142 (16.2)			36 (7.2)	33 (4.3)	69 (5.5)		25 (3.4)	31 (2.8)	56 (3.0)	
Morbidities^a^, n (%)													
Hypertension	120 (32.0)	158 (31.7)	278 (31.8)	0.916		213 (42.4)	447 (58.7)	660 (52.3)	< 0.001	448 (60.3)	746 (67)	1194 (64.3)	0.003
Heart problem	208 (55.5)	259 (51.9)	467 (53.4)	0.296		258 (51.4)	431 (56.6)	689 (54.6)	0.067	384 (51.7)	582 (52.3)	966 (52.0)	0.797
Stroke	38 (10.1)	31 (6.2)	69 (7.9)	0.033		34 (6.8)	33 (4.3)	67 (53.0)	0.060	62 (8.3)	68 (6.1)	130 (7.0)	0.065
Diabetes	48 (12.8)	47 (9.4)	95 (10.9)	0.112		51 (10.2)	98 (12.9)	149 (11.8)	0.143	149 (20.1)	198 (17.8)	347 (18.7)	0.220
Osteoarthritis	119 (31.7)	194 (38.9)	313 (35.8)	0.029		188 (37.5)	356 (46.8)	544 (43.1)	0.001	311 (41.9)	515 (46.3)	826 (44.5)	0.061
Hip fracture	75 (20.0)	77 (15.4)	152 (17.4)	0.078		94 (18.7)	123 (16.2)	217 (17.2)	0.237	141 (19.0)	132 (11.9)	273 (14.7)	< 0.001
Depression	123 (32.8)	85 (17)	208 (23.8)	< 0.001		142 (28.3)	97 (12.9)	239 (19.0)	0.001	197 (26.5)	111 (10.0)	308 (16.6)	< 0.001
Number of morbidities^a^													
0	43 (11.5)	76 (15.2)	119 (13.6)	0.016		68 (13.5)	69 (9.1)	137 (10.8)	0.004	77 (10.4)	90 (8.1)	167 (9.0)	< 0.001
1	96 (25.6)	164 (32.9)	260 (29.7)			137 (27.3)	178 (23.4)	315 (24.9)		137 (18.4)	259 (23.3)	396 (21.3)	
2	124 (33.1)	135 (27.1)	259 (29.6)			138 (27.5)	249 (32.7)	387 (30.6)		215 (28.9)	361 (32.4)	576 (31.0)	
3	69 (18.4)	86 (17.2)	155 (17.7)			88 (17.5)	174 (22.9)	262 (20.7)		179 (24.1)	270 (24.3)	449 (24.2)	
> = 4	43 (11.5)	38 (7.6)	81 (9.3)			71 (14.1)	91 (12.0)	162 (12.8)		135 (18.2)	133 (11.9)	268 (14.4)	
*Mobility^a^ disability	259 (71.2)	238 (48.2)	497 (57.9)	< 0.001		383 (77.4)	360 (48.7)	743 (60.2)	< 0.001	522 (70.9)	441 (40.7)	963 (52.9)	< 0.001
**ADL disability^a^	174 (47.0)	75 (15.2)	249 (28.8)	< 0.001		233 (46.8)	73 (9.6)	306 (24.4)	< 0.001	312 (42.2)	87 (7.9)	399 (21.7)	< 0.001

Notes *Mobility disability = dependent in at least one activity among moving indoors, walking 400 m and climbing stairs

^**^ADL disability = dependent in at least one activity among dress and undress and get in and out of bed. *SD* Standard deviation

^a^Pearson’s chi-square test

^b^Mann-Whitney U-test

### Association between dementia and disability

People with dementia had higher odds for ADL disability (OR = 7.29, 95% CI = 6.15–8.64) compared to those without dementia when adjusted for age, gender, and study year (Table 2, model 1). This barely changed after adjusting for occupational class and number of comorbidities (models 2–3) (OR = 7.19, 95% CI = 6.05–8.53). People with dementia were more likely to have mobility disability than those without dementia after adjusting for age, gender, and study year (OR 3.35, 95% CI = 2.90–3.86) (Table 3, model 1). This association did not change when occupational class and number of chronic conditions were taken into account (model 2–3) (OR 3.34, 95% CI = 2.89–3.87). The OR showing the association with dementia was much higher for ADL disability than for mobility disability. Moreover, based on PAF for all the years together, 56.6% (95% CI = 52.1–60.6) of ADL disability and 18.1% (95% CI = 15.9–20.2) of mobility disability were attributable to dementia.

**Table 2:** Association between dementia and ADL disability among the oldest old in 2001, 2010, and 2018

**Variables**	Model 1		Model 2		Model 3		Model 4	
	OR (95% CI)	*p*	OR (95% CI)	*p*	OR (95% CI)	*p*	OR (95% CI)	*p*
**Dementia (ref = no dementia)**	7.29 (6.15–8.64)	< 0.001	7.20 (6.07–8.53)	< 0.001	7.19 (6.05–8.53)	< 0.001	4.59 (3.30–6.39)	< 0.001
**Age**	1.13 (1.09–1.16)	< 0.001	1.13 (1.10–1.16)	< 0.001	1.13 (1.10–1.17)	< 0.001	1.13 (1.10–1.17)	< 0.001
**Gender (ref = male) Female**	1.55 (1.25–1.91)	< 0.001	1.53 (1.23–1.89)	< 0.001	1.44 (1.16–1.78)	0.001	1.44 (1.16–1.78)	< 0.001
**Study year (ref = 2001)**								
**2010**	0.78 (0.63–0.97)	0.025	0.86 (0.69–1.07)	0.182	0.83 (0.66–1.04)	0.107	0.58 (0.41–0.83)	0.002
**2018**	0.66 (0.54–0.81)	< 0.001	0.74 (0.60–0.92)	0.007	0.69 (0.56–0.86)	0.001	0.47 (0.34–0.66)	< 0.001
**Occupational class (ref = Non-manual)**								
**Manual**			0.97 (0.81–1.16)	0.741	0.94 (0.79–1.13)	0.505	0.93 (0.78–1.12)	0.463
**Housewives**			0.92 (0.66–1.28)	0.630	0.93 (0.66–1.29)	0.649	0.92 (0.66–1.29)	0.642
**Unknown**			2.28 (1.67–3.13)	< 0.001	2.28 (1.66–3.14)	< 0.001	2.30 (1.68–3.14)	< 0.000
**Number of morbidities 0 (ref)**								
**1**					0.94 (0.70–1.28)	0.698	0.95 (0.70–1.29)	0.739
**2**					0.90 (0.67–1.21)	0.496	0.93 (0.69–1.24)	0.611
**3**					1.26 (0.93–1.71)	0.135	1.29 (0.95–1.75)	0.103
≥ **4**					1.85 (1.33–2.56)	< 0.001	1.88 (1.36–2.61)	< 0.001
Dementia * study year (ref = 2001)								
Dementia * 2010							1.80 (1.15–2.83)	0.010
Dementia * 2018							1.85 (1.21–2.83)	0.005

ADL disability = dependent in at least one activity among dress and undress and get in and out of bed. Outcome variable is ADL disability in all models

The explanatory variables are as follows

Model 1 – dementia, age, gender, and study year

Model 2 – dementia, age and gender, study year, and occupational class

Model 3 – dementia, age, gender, study year, occupational class, and multimorbidity

Model 4 – dementia, age, gender, study year, occupational class, multimorbidity, and interaction between dementia and study year

**Table 3:** Association between dementia and mobility disability among the oldest old in 2001, 2010, and 2018

**Variables**	Model 1		Model 2		Model 3		Model 4	
	OR (95% CI)	*p*	OR (95% CI)	*p*	OR (95% CI)	*p*	OR (95% CI)	*p*
**Dementia (ref = no dementia)**	3.35 (2.90–3.86)	< 0.001	3.28 (2.84–3.78)	< 0.001	3.34 (2.89–3.87)	< 0.001	2.42 (1.79–3.28)	< 0.001
**Age**	1.17 (1.14–1.20)	< 0.001	1.17 (1.14–1.20)	< 0.001	1.17 (1.14–1.21)	< 0.001	1.17 (1.14–1.21)	< 0.001
**Gender (ref = male) Female**	2.44 (2.07–2.88)	< 0.001	2.45 (2.07–2.89)	< 0.001	2.21 (1.87–2.62)	< 0.001	2.21 (1.87–2.62)	< 0.001
**Study year (ref = 2001)**								
**2010**	1.10 (0.91–1.33)	0.314	1.15 (0.95–1.40)	0.155	1.06 (0.87–1.29)	0.582	0.90 (0.71–1.15)	0.404
**2018**	0.82 (0.69–0.98)	0.030	0.87 (0.72–1.04)	0.129	0.75 (0.62–0.90)	0.002	0.64 (0.51–0.81)	< 0.001
**Occupational class (ref = Non-manual)**								
**Manual**			1.33 (1.15–1.54)	< 0.001	1.25 (1.08–1.46)	0.003	1.25 (1.08–1.46)	0.004
**Housewives**			1.04 (0.79–1.37)	0.793	1.02 (0.77–1.35)	0.913	1.01 (0.76–1.35)	0.920
**Unknown**			1.48 (1.09–2.01)	0.012	1.48 (1.09–2.02)	0.013	1.50 (1.10–2.05)	0.010
**Number of morbidities 0 (ref)**								
**1**					1.35 (1.05–1.75)	0.021	1.36 (1.05–1.76)	0.019
**2**					1.80 (1.40–2.31)	< 0.001	1.83 (1.43–2.35)	< 0.001
**3**					2.54 (1.95–3.31)	< 0.001	2.58 (1.98–3.36)	< 0.001
≥ **4**					4.89 (3.58–6.67)	< 0.001	4.96 (3.63–6.77)	< 0.001
Dementia * study year (ref = 2001)								
Dementia * 2010							1.56 (1.04–2.33)	0.033
Dementia * 2018							1.50 (1.04–2.17)	0.032

Mobility disability = dependent in at least one activity among moving indoors, walking 400 m, and climbing stairs. Outcome variable is mobility disability in all models

The explanatory variables are as follows

Model 1 – dementia, age, gender, and study year

Model 2 – dementia, age and gender, study year, and occupational class

Model 3 – dementia, age, gender, study year, occupational class, and multimorbidity

Model 4 – dementia, age, gender, study year, occupational class, multimorbidity, and interaction between dementia and study year

**Fig. 1:**
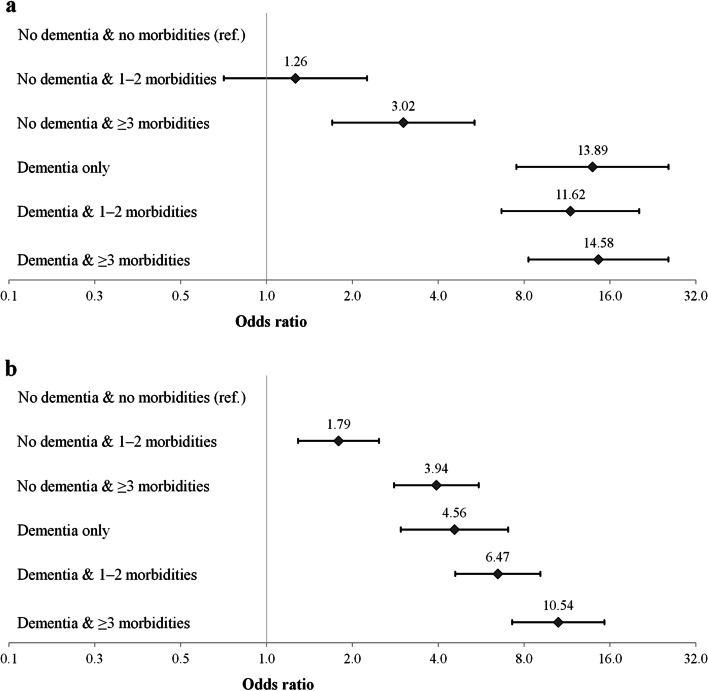
Association of dementia and comorbidity with (a) ADL disability and (b) mobility disability among the oldest old in 2001, 2010 and 2018 combined

The combined effect of dementia and comorbidities on ADL and mobility disability is illustrated in Fig. 1. The participants were categorized into six groups, and the reference group consisted of those who did not have dementia or any other morbidity (7.1%). For ADL disability, in people with no dementia but three or more other conditions the OR increased according to the number of morbidities and was 3.02 (95% CI = 1.6–5.4). In people with dementia but no other conditions, the likelihood of ADL disability was clearly higher (OR = 13.9, 95% CI = 7.4–24.9) than among those with three or more other conditions but no dementia (3.02, 95% CI = 1.6–5.4). The number of comorbidities with dementia did not increase the likelihood of ADL disability. The likelihood of mobility disability increased according to the number of morbidities among people with and without dementia. In the group with only dementia, the odds of having mobility disability was 4.56 (95% CI = 2.9–7.0), approximately the same as in the group with three morbidities without dementia, and it increased gradually with comorbidities, being 10.5 (95% CI = 7.2–15.3) in the group with dementia and at least three comorbidities.

### Differences over time in association between dementia and disability (interaction model)

**Fig. 2:**
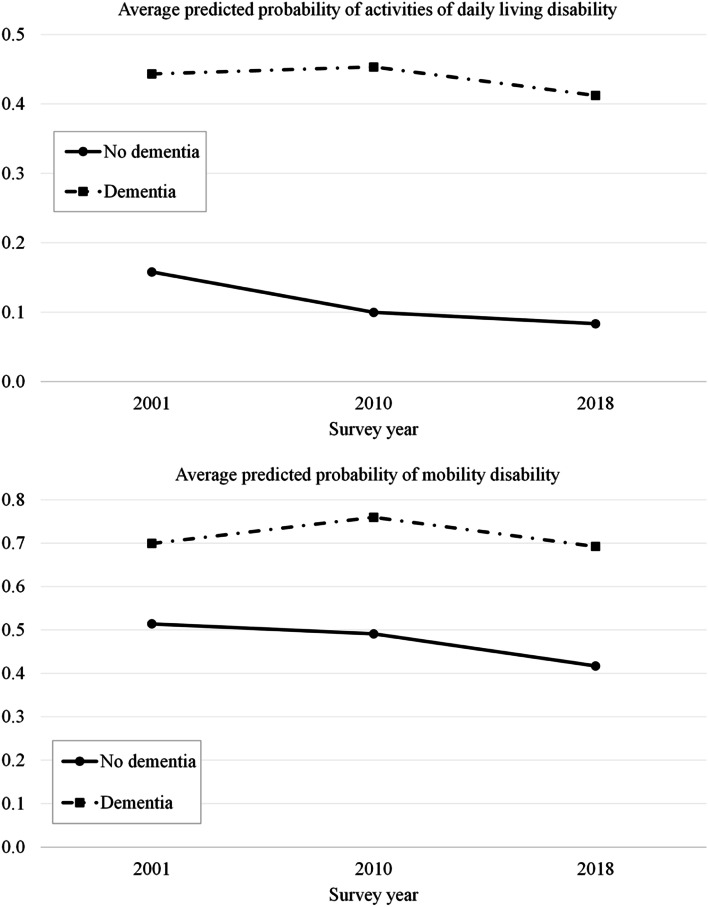
Average predicted probability of activities of daily living and mobility disability in people with and without dementia over time (based on model 4 in Tables 2 and 3)

Interaction between dementia status and study year was statistically significant (Table 2, model 4), i.e., the effect of dementia on ADL disability was higher in 2010 (OR 1.80, 95% CI = 1.15–2.83) and 2018 (OR 1.85, 95% CI = 1.21–2.83) than in 2001. ADL disability decreased in people with and without dementia. In later study years, ADL disability was largely concentrated in people with dementia (Fig. 2). The time trend was rather similar for mobility as for ADL disability: the effect of dementia on mobility disability changed significantly over the study years and was greater in 2010 (OR 1.56, 95% CI = 1.04–2.33) and in 2018 (OR 1.50, 95% CI = 1.04–2.17) than in 2001. Mobility disability decreased in people with and without dementia, but as this decrease was greater in the latter group, the difference in disability between the groups increased over time (Fig. 2). The results for both ADL and mobility disability are presented separately for each year in supplementary tables (Additional file 1).

## Discussion

Using data from repeated identical cross-sectional surveys in 2001, 2010, and 2018, we add to the limited research on the connection between dementia and functional disability in the growing population of persons aged 90 and over. We found a strong association between dementia and disability in ADL and mobility even after controlling for age, gender, occupational class, number of chronic conditions, and study year. Functional ability has improved over time in the oldest old [10], but according to our results it improved more in people without dementia than in those with dementia. In addition, very old people with only dementia (no comorbidity) had clearly a higher ADL disability and also somewhat higher mobility disability than those without dementia but with up to three or more morbidities. These results show that dementia plays a major role in explaining functional disability among the oldest old*.* This suggests, importantly, that the increase in the number of people with dementia means not only a larger number of people with cognitive problems, but also a larger number of people with physical disabilities.

To our knowledge, this is the first population-based study examining the role of comorbidities in the association between dementia and functional disability and exploring how functional disability in people with dementia has changed over time in this very old age group.

Dementia is the leading cause of disability in old age. The disability pathways depend on age and several internal and external factors, such as the type of disease causing dementia, comorbidities, and available medical and rehabilitative services.

[30]. Ageing-related decline in cognitive ability and mobility has been explained by neurological degeneration, inflammation and damaged vasculature [31]. Although robust evidence is available on age-related changes in physical ability in the oldest old [32–34], there has been only limited research into their connections with dementia. Our results are consistent with several cross-sectional [35, 36] and longitudinal studies [37, 38] among younger old people and oldest old [39, 40], which have reported excess disability in people with dementia compared to those without, even after taking into account the number of chronic conditions.

Our findings show that the difference in disability between those with and without dementia increased over the study period from 2001 to 2018. It has been suggested that better living conditions, improved cognitive ability, early diagnosis of dementia, improved medical care, and physical and technological support can contribute to improved functional ability in older people [8, 9]. However, our findings suggest that these improvements are mainly seen in people without dementia. Although it has been reported that functional dependence is associated with multimorbidity [12, 41], in our study dementia comorbidity did not increase disability in ADL, which was high even without additional conditions, but it did increase mobility disability. It is important to stress that ADL disability is measured in different ways in different studies. We measured ADL disability with two variables (dress and undress and get in and out of bed), which reflect severe disability.

Vitality 90 + is a unique study that has used a similar design and the same measurements across a number of years to examine total populations including both community-living and institutionalized persons aged 90 and over. The inclusion of institutionalized persons is rare in this line of research but facilitates unbiased estimates of health problems in very old people [42]. The sample sizes were large and response rates high in all study years.

Studying dementia-related disability in the oldest old involves several challenges. The data for our study was collected using self-reports, the method of choice in numerous population-based studies in older adults and among the oldest old [9, 33, 43]. For both practical and financial reasons the only viable option for population-based studies, and repeated population-based studies in particular, is the survey methodology. To receive representative population-based information on the oldest-old, proxy respondents are necessary [42]. In our study, the proportion of proxy participants varied from 15 to 23%, and among those with dementia, from 34 to 47%, depending on the year of data collection. The sensitivity analyses conducted separately for self-respondents and proxy respondents (not presented) show that the basic patterns of associations between multimorbidity and disability are largely similar: in both groups, dementia alone was associated with higher or at least as high ADL disability than 3 or more morbidities without dementia. Also in both respondent groups, among those with dementia, additional morbidities increased the likelihood of mobility disability, but not that of ADL disability. The experience from our study as well as from several others suggests that if proxy responses are accepted and if the questionnaire is clear and not too long, surveys are a feasible method of data collection among the oldest old [44]. Furthermore, survey data on health and functioning among persons aged 90 and over has reasonable validity and reliability [10, 25 42].

In our study, mobility and ADL were assessed using simple and easy-to-answer questions. For mobility disability, we used a standard and validated set of questions [45]. In the interest of feasibility and a high response rate among the oldest-old, we chose to use only two ADL items that are part of a validated and widely used ADL scale [45]. The major role of these two questions in the longer ADL scale has been verified in another sample [46], and its logical behaviour is confirmed in numerous earlier analyses in the Vitality 90 + study.

We understand that cognitive impairment may hamper the reliability of self-reported data. However, previous studies have reported that people in the mild stages of dementia are able to convey reliable information regarding their health [47, 48]. Also, self-reports are often thought to underestimate medical conditions. Yet Goebeler et al. (2007) found that in earlier rounds of the Vitality 90 + study, the prevalence of self-reported doctor-diagnosed dementia and depression was higher than indicated by the medical records, probably partly because of the wording of the question [43]. Self-reported information on chronic conditions was quite consistent with medical records, although discrepancies were higher in persons over 90 years in the Vitality 90 + Study compared to other studies in younger olds [43]. In the present study, around 40% of the answers were given by proxies for persons with dementia. Proxies were mainly used for very frail individuals, mostly in the severe stages of dementia who otherwise would not have been able to participate, while those with better health responded themselves. Several researchers have reported that proxies can be considered reliable reporters and response comparability can be improved by objective, observable, or easy questions [49, 50]. In all, we believe that, given all the uncertainties associated with self-reported data, our findings add valuable information about disability in the oldest old with dementia.

The very high PAF for ADL and mobility disability attributed to dementia is a critical policy issue for health and social care planners [38]. As the number of people with dementia continues to rise, so does the number of individuals with functional disability, which in turn likely lead to increased dependency and an increased need for institutional care. Especially the combination of dementia, comorbidities, and high disability emphasizes the need for individually planned person-centered care [16], as multiple needs are otherwise challenging to recognize and meet.

There is a need for interventions aimed specifically at people with cognitive impairment and dementia in order to try and maintain their functional ability [51]. In addition, the connection between dementia and functional ability needs to be taken into account in national ageing and care policy-making as people with dementia appear to have major challenges in physical functioning in addition to cognitive abilities. Care and rehabilitation should be optimized in view of the heterogeneity in disability and treatment goals [6]. Sufficient professional support relevant for rehabilitation to meet the complex needs of people with dementia should be included in the care pathway [52].

## Conclusion

Dementia is the main single contributor to physical disability in old age that seems to have continued to gain in importance over time. Dementia increases the probability of functional disability among the oldest old, and an increasing number of comorbidities further increases the likelihood of mobility disability. The future prevalence of dementia will have a significant impact on the number of people with physical impairments, and therefore interventions aimed at preventing or delaying dementia and the treatment of dementia will have a crucial role in promoting the mobility and functioning of older people. However, it is also necessary to introduce new approaches in clinical practice, rehabilitative services, and care in order to maintain functional capacity and delay ADL disability and impaired mobility in people who are already diagnosed with dementia. In addition, further research is needed to elucidate the mechanisms of dementia-related disability in order to introduce new treatment and prevention pathways.

## Supplementary Information

**Additional file 1:** **Supplementary Table 1.** Association between dementia and ADL disability among the oldest old separately in 2001, 2010, and 2018. **Supplementary Table 2.** Association between dementia and mobility disability among the oldest old separately in 2001, 2010 and 2018.

## Data Availability

Vitality 90 + data sets are described in a public, open access repository (Finnish Social Science Data Archive) and data are available upon reasonable request. Requests to access the datasets should be directed to Linda Enroth, linda.enroth@tuni.fi. Permanent links to datasets: 2014 https://urn.fi/urn:nbn:fi:fsd:T-FSD3013, 2010 https://urn.fi/urn:nbn:fi:fsd:T-FSD3012

## Abbreviations

ADL: Activities of daily living
GEE: Generalized estimating equation
CI: Confidence interval
OR: Odds ratio
PAF: Population attributable fraction
