# Supplementary material for: Dementia-related disability in the population aged 90 years and over: differences over time and the role of comorbidity in the vitality 90 + study
Source: BMC Geriatr. 2023 May 6;23:276. doi: 10.1186/s12877-023-03980-5 (PMC10163713; doi:10.1186/s12877-023-03980-5)
Supplement: Supplementary file 1 — Additional file 1: Supplementary Table 1. Association between dementia and ADL disability among the oldest old separately in 2001, 2010, and 2018. Supplementary Table 2. Association between dementia and mobility disability among the oldest old separately in 2001, 2010 and 2018. [file 12877_2023_3980_MOESM1_ESM.docx]

**Additional file**

**File name**: Additional file 1

**File format**: Word document

**Title of data**

Supplementary Table 1: Association between dementia and ADL disability among the oldest old separately in 2001, 2010, and 2018

Supplementary Table 2: Association between dementia and mobility disability among the oldest old separately in 2001, 2010 and 2018

Description of data: Supplementary tables showing results.

Supplementary Table 1: Association between dementia and ADL disability among the oldest old separately in 2001, 2010, and 2018 (adjusted odds ratios (OR) and 95% confidence intervals (CI) from binary logistic regression models)

| **Variables** | 2001 |  | 2010 |  | 2018 |  |
| --- | --- | --- | --- | --- | --- | --- |
|  | OR (95% CI) | *p* | OR (95% CI) | *p* | OR (95% CI) | *p* |
| **Dementia (ref = no dementia)** | 4.68 (3.33–6.57) | <0.001 | 8.04 (5.90–10.96) | <0.001 | 8.59 (6.56–11.26) | <0.001 |
| **Age** | 1.13 (1.06–1.21) | <0.001 | 1.15 (1.09–1.21) | <0.001 | 1.12 (1.07–1.17) | <0.001 |
| **Gender (ref = male)**  **Female** | 1.54 (0.97–2.45) | 0.066 | 1.65 (1.08–2.52) | 0.021 | 1.29 (0.96–1.74) | 0.096 |
| **Occupational class**  **(ref = Non-manual)** |  |  |  |  |  |  |
| **Manual** | 0.65 (0.44–0.97) | 0.036 | 1.19 (0.86–1.65) | 0.300 | 0.94 (0.72–1.22) | 0.628 |
| **Housewives** | 0.37 (0.19–0.72) | 0.004 | 1.17 (0.68–2.03) | 0.571 | 1.65 (0.95–2.87) | 0.077 |
| **Unknown** | 1.74 (1.07–2.85) | 0.026 | 2.81 (1.55–5.11) | <0.001 | 1.93 (0.97–3.82) | 0.600 |
| **Number of morbidities**  **0 (ref = 0)** |  |  |  |  |  |  |
| **1** | 1.69 (0.89–3.19) | 0.109 | 0.69 (0.42–1.15) | 0.158 | 0.93 (0.57–1.51) | 0.758 |
| **2** | 2.41 (1.29–4.50) | 0.006 | 0.72 (0.44–1.18) | 0.192 | 0.65 (0.41–1.04) | 0.070 |
| **3** | 3.58 (1.84–6.93) | <0.001 | 0.68 (0.40–1.17) | 0.165 | 1.10 (0.69–1.76) | 0.688 |
| ≥**4** | 6.49 (3.09–13.65) | <0.001 | 1.28 (0.73–2.23) | 0.390 | 1.33 (0.81–2.18) | 0.264 |

Notes: ADL disability = dependent in at least one activity among dress and undress and get in and out of bed. Outcome variable is ADL disability and explanatory variables are dementia, age, gender, occupational class, and multimorbidity

Supplementary Table 2: Association between dementia and mobility disability among the oldest old separately in 2001, 2010 and 2018 (adjusted odds ratios (OR) and 95% confidence intervals (CI) from binary logistic regression models)

| **Variables** | 2001 | | 2010 | | 2018 | |
| --- | --- | --- | --- | --- | --- | --- |
|  | OR (95% CI) | *p* | OR (95% CI) | *p* | OR (95% CI) | *p* |
| **Dementia (ref = no dementia)** | 2.35 (1.73–3.20) | <0.001 | 3.78 (2.87–4.98) | <0.001 | 3.64 (2.94–4.51) | <0.001 |
| **Age** | 1.22 (1.14–1.30) | <0.001 | 1.21(1.15–1.28) | <0.001 | 1.14 (1.10–1.19) | <0.001 |
| **Gender (ref = male)**  **Female** | 1.89 (1.29–2.77) | 0.001 | 2.52 (1.82–3.49) | <0.001 | 2.20 (1.73–2.79) | <0.001 |
| **Occupational class**  **(ref = Non-manual)** |  |  |  |  |  |  |
| **Manual** | 1.17 (0.82–1.67) | 0.376 | 1.39 (1.06–1.82) | 0.018 | 1.19 (0.96–1.48) | 0.106 |
| **Housewives** | 0.68 (0.40–1.16) | 0.158 | 1.12 (0.68–1.84) | 0.650 | 1.39 (0.86–2.24) | 0.178 |
| **Unknown** | 1.71 (1.05–2.79) | 0.031 | 0.97 (0.53–1.75) | 0.908 | 1.68 (0.92–3.04) | 0.089 |
| **Number of morbidities**  **0 (ref = 0)** |  |  |  |  |  |  |
| **1** | 1.35 (0.84–2.18) | 0.216 | 1.21 (0.77–1.91) | 0.403 | 1.49 (0.99–2.25) | 0.056 |
| **2** | 2.86 (1.76–4.66) | <0.001 | 1.73 (1.11–2.70) | 0.015 | 1.55 (1.05–2.30) | 0.028 |
| **3** | 3.18 (1.86–5.45) | <0.001 | 1.98 (1.24–3.17) | 0.005 | 2.75 (1.83–4.13) | <0.001 |
| ≥**4** | 5.00 (2.50–9.97) | <0.001 | 4.95 (2.81–8.72) | <0.001 | 4.81 (3.03–7.61) | <0.001 |

Notes: Mobility disability = dependent in at least one activity among moving indoors, walking 400 m, and climbing stairs. Outcome variable is mobility disability and explanatory variables are dementia, age, gender, occupational class, and multimorbidity.
